# Supplementary material for: Molecular Characterization of Three Tandemly Located Flagellin Genes of Stenotrophomonas maltophilia
Source: Int J Mol Sci. 2022 Mar 31;23(7):3863. doi: 10.3390/ijms23073863 (PMC8998449; doi:10.3390/ijms23073863)
Supplement: Supplementary file 1 [file ijms-23-03863-s001.zip › ijms-1595070-supplementary.pdf]

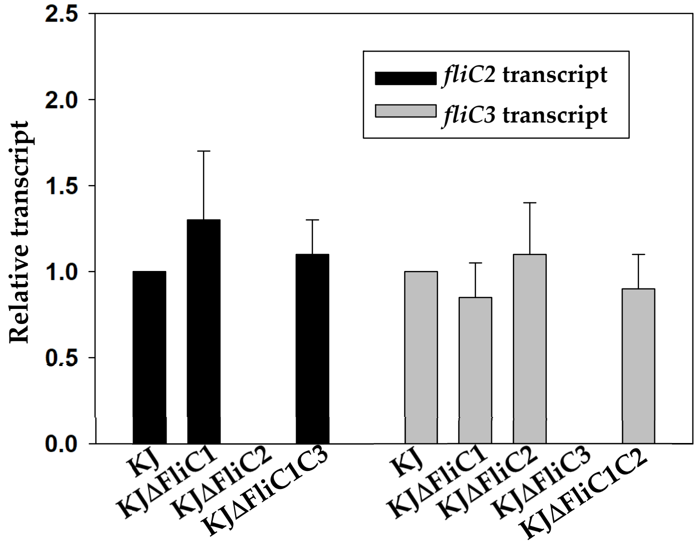

Figure S1. Evaluation of polar effect in the *fliC*-associated mutants. Overnight-cultured *S. maltophilia* tested was inoculated into fresh LB broth at an initial OD_{450nm} of 0.15 and then further cultured for 5 h. The *fliC2* and *fliC3* transcripts of the strains indicated were quantified by qRT-PCR. Bars represent the mean from three independent experiments. Error bars represent the standard error (SD) of the mean. Data are expressed as mean ± SD.

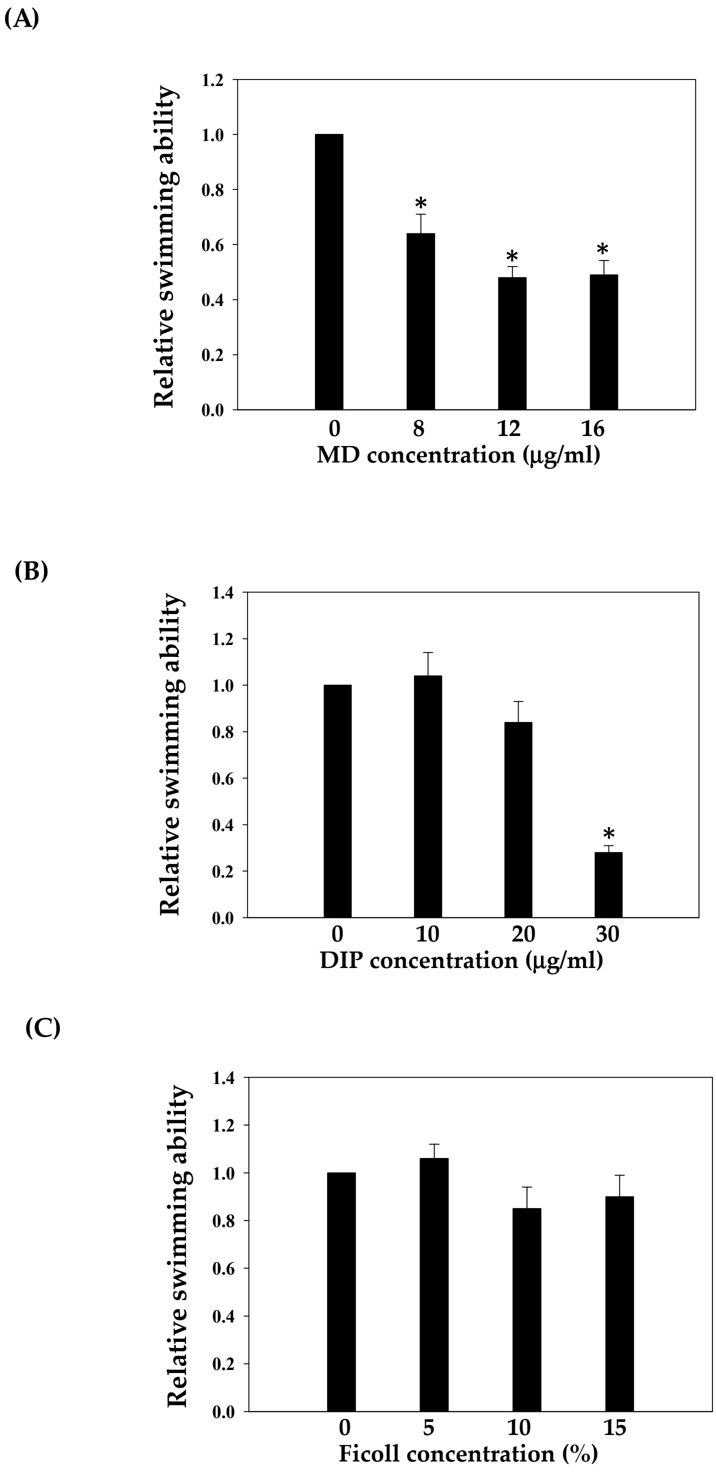

Figure S2. The impact of MD, DIP, and Ficoll on swimming motility. Five microliters of overnight-cultured *S. maltophilia* KJ cells were inoculated into swimming agar without and with additives of MD (A), DIP (B), and Ficoll (C), and then incubated at 37°C for 48 h. Swimming zones were recorded. The relative swimming motility was normalized to the condition of no additive. Data are the means from three independent experiments. Error bars indicate the standard deviations for three triplicate samples. *, $P < 0.05$, significance calculated by Student's *t* test.

Table S1 The numbers of flagellin gene in different genogroups of *S. maltophilia* complex

Strain	Genogroup	No. of flagellin gene
1189_SMAL	1	1
ISMMS6	1	2
ISMMS7	1	3
30_SMAL	2-a	2
53	2-a	2
C357	2-a	3
H_22_creteil	2-a	2
SJTL3	2-b	3
AU12-09	3	3
910	4	3
R551-3	5	3
RA8	5	3
K279a	6	3
WJ66	6	3
EPM1	6	3
259_SMAL	7	1
G51	7	3
5BA-1-2	9-a	3
EP20	9-b	3
SM-3123	A	3
EA1	B	3
D457	C	3
M30	C	3
ISMMS3	D	3
197_SMAL	F	1
22_SMAL	F	2
E759	F	3
MS_2009	G	3
MF89	H	2
ATCC19867	H	3
JV3	I	3
H_69_creteil	J	2
E301	J	3
AA1	Smc_1	2
LMG 978	Single	3

Table S2. Bacterial strains, plasmids, and primers used in this study

Strain, plasmid, or primer	Genotype or properties	Reference
S. maltophilia		
KJ	A clinical *S. maltophilia* isolate	1
KJΔFliC1 KJΔFliC2	*S. maltophilia* KJ mutant of *fliC1* gene; *ΔfliC1*	This study
	S. maltophilia KJ mutant of *fliC2* gene; *ΔfliC2*	This study
KJΔFliC3	*S. maltophilia* KJ mutant of *fliC3* gene; *ΔfliC3*	This study
KJΔFliC2C3	*S. maltophilia* KJ mutant of *fliC2* and *fliC3* genes; *ΔfliC2*, *ΔfliC3*	This study
KJΔFliC1C3	*S. maltophilia* KJ mutant of *fliC1* and *fliC3* genes; *ΔfliC1*, *ΔfliC2*	This study
KJΔFliC1C2	*S. maltophilia* KJ mutant of *fliC1* and *fliC2* genes; *ΔfliC1*, *ΔfliC2*	This study
KJΔFliC1C2C3	*S. maltophilia* KJ mutant of *fliC1*, *fliC2*, and *fliC3* genes; *ΔfliC1*, *ΔfliC2*, *ΔfliC3*	This study
E. coli		
DH5a	F- φ80d*lacZΔM15* Δ(*lacZYA-argF*)*U169 deoR recA1 endA1 hsdR17* (r_K^- m_K^+) *phoA supE44λ thi-1 gyrA96 relA1*	Invitrogen
S17-1	λ*pir*$^+$ mating strain	
**Plasmids**		
pEX18Tc	*sacB oriT*, Tc^r	3
pRKXylE	Plasmid pRK415 containing a *xylE* cassette and the orientation of *xylE* gene being opposite to the orientation of *lacZ* promoter of pRK415; Tc^r	4
pΔFliC1	pEX18Tc with an internal-deletion *fliC1* gene; Tc^r	This study
pΔFliC2	pEX18Tc with an internal-deletion *fliC2* gene; Tc^r	This study
pΔFliC3	pEX18Tc with an internal-deletion *fliC3* gene; Tc^r	This study
pFliC1$_{xylE}$	pRK415 with a P_{fliC1}-*xylE* promoter transcriptional fusion; Tc^r	This study
pFliC2$_{xylE}$	pRK415 with a P_{fliC2}-*xylE* promoter transcriptional fusion; Tc^r	This study
pFliC3$_{xylE}$	pRK415 with a P_{fliC3}-*xylE* promoter transcriptional fusion; Tc^r	This study
**Primers**		
FliC1N-F	5′- CTGAGCTCGTACCAGCTGCA -3′	This study
FliC1N-R	5′- GAGGATCCAGCGACATCGTA - 3′	This study
FliC1C-F	5′- CCGGATCCAAACCAGCACCA -3′	This study
FliC1C-R	5′- GCTCTAGATACGCTGAGCAT -3′	This study
FliC2C-F	5′- CGTCTAGAAGATCGACGTAT -3′	This study
FliC2C-R	5′- TGGCATGCGCATTCAACGAC -3′	This study
FliC3C-F	5′- AAGCATGCCGCAGAACTGA -3′	This study
FliC3C-R	5′- CAAAGCTTGCCATGTGATTCT -3′	This study
FliC3-C	5′- CTTGTCGGCGAAGCTCTTG-3′	This study
FliC1Q178-F	5′- CAGCGATCAACAACAAGCTG-3′	This study
FliC1Q178-R	5′- GTGCTGGTTTCGGTCAGG-3′	This study
FliC2Q163-F	5′- TCATATCAACGCCAAGATCG-3′	This study
FliC2Q163-R	5′- GGTCACGTCGTCGGTGTAG-3′	This study
SmeXQ-F	5′-TACGACCGCCGCAAGCAACC-3′	4
SmeXQ-R	5′- CAGCTCGAAGTAGTTGCGTGCC-3′	4
rDNA-F	5′- GACCTTGCGCGATTGAATG -3′	4
rDNA-R	5′- CGGATCGTCGCCTTGGT -3′	4

1. Hu RM, Huang KJ, Wu LT, Hsiao YJ, Yang TC. 2008. Induction of L1 and L2 beta-lactamases of *Stenotrophomonas maltophilia*. Antimicrob Agents Chemother 52:1198-1200.

2. Chen CH, Huang CC, Chung TC, Hu RM, Huang YW, Yang TC. 2011. Contribution of resistance-nodulation-division efflux pump operon *smeU1-V-W-U2-X* to multidrug resistance of *Stenotrophomonas maltophilia*. Antimicrob. Agents Chemother 55:5826-5833.
